# Diagnostic information in GP referral letters to a memory clinic: a retrospective cohort study

**DOI:** 10.3399/BJGPO.2024.0065

**Published:** 2025-02-12

**Authors:** Demi Ronner, Dorien Oostra, Jurgen Claassen, Edo Richard, Marieke Perry

**Affiliations:** 1 Department of Primary and Community Care, Radboud University Medical Center (UMC) Alzheimer Center, Radboud UMC, Nijmegen, Netherlands; 2 Department of Geriatric Medicine, Donders Institute for Brain, Cognition and Behavior, Radboud UMC, Nijmegen, Netherlands; 3 Department of Cardiovascular Sciences, University of Leicester, Leicester, UK; 4 Department of Neurology, Radboud UMC Alzheimer Center, Radboud UMC, Nijmegen, Netherlands; 5 Department of Public and Occupational Health, Amsterdam Public Health Research Institute, Amsterdam UMC, location Academic Medical Center, Amsterdam, Netherlands

**Keywords:** dementia, diagnosis, care of older people, aged

## Abstract

**Background:**

Dementia diagnostics can often be performed in primary care, yet older people with memory complaints are frequently referred to memory clinics (MCs).

**Aim:**

To compare diagnostic information in GP referral letters of patients with and without an eventual dementia diagnosis.

**Design & setting:**

Retrospective cohort study in a Dutch academic MC.

**Method:**

We collected electronic health record (EHR) data of consecutive patients aged ≥65 years referred by their GP between 2016 and 2020. EHR data included patient characteristics, diagnostic information in referral letters, ancillary investigations performed at the MC, and established diagnoses. We performed χ^2^ tests to compare groups.

**Results:**

Of 651 patients included, the average age was 78.0 years (standard deviation 6.8) and 348 (53.5%) were diagnosed with dementia. Most people with dementia were diagnosed without ancillary investigations (*n* = 235/348, 67.5%). In GP referral letters of people with dementia compared with people without dementia, a collateral history, any physical examination, a differential diagnosis including dementia, a Mini-Mental State Examination score, interference with daily functioning, and decline from previous levels of functioning were mentioned more often. Furthermore, the more diagnostic criteria mentioned in the referral letter, the more often dementia was diagnosed at the MC (no criteria: 35.4%; one criterion: 47.3%; two criteria: 53.4%; three criteria: 69.9%; and four or five criteria: 83.3%).

**Conclusion:**

GPs often correctly mention diagnostic information and dementia criteria in referral letters of people with dementia, and they are often diagnosed without ancillary investigations. This suggests that referral is often unnecessary, and GPs can be empowered to diagnose dementia themselves.

## How this fits in

Dutch dementia guidelines encourage diagnosing dementia in primary care, but >60% of dementia diagnoses are currently established in a memory clinic (MC). Given the expected rise in the number of people with dementia, diagnosing in primary care whenever possible will become increasingly important. This study shows that GPs often implicitly diagnose dementia correctly by mentioning criteria for dementia in their referral letters and that nearly two-thirds of older people with dementia do not require ancillary investigations at an MC. This underlines the fact that dementia is a clinical diagnosis and suggests that more patients could be diagnosed in primary care.

## Introduction

Dementia is a clinical diagnosis based on cognitive impairment of sufficient severity to interfere with daily activities.^1,2^ Either a GP or a medical specialist can establish the diagnosis.^3,4^ Diagnosing in primary care whenever possible is essential to maintain the accessibility and affordability of memory clinic (MC) services, especially considering the increasing waiting times in the UK^5^ and the Netherlands,^6,7^ and the expected increase in people with dementia in the coming years.^8^ GPs are in an ideal position to observe and interpret changes in their patients' cognitive and functional abilities, owing to their long-term relationships with patients and understanding of the patient’s social context. Although Dutch GP guidelines encourage a primary care diagnosis,^3^ specialists in hospital-based MCs establish around 60% of dementia diagnoses in the Netherlands.^9,10^ Several possible explanations exist for this discrepancy between guideline recommendations and daily practice.

Throughout the years, GPs have consistently reported barriers in diagnosing patients in primary care, including a perceived lack of knowledge or training, time and resources, and diagnostic uncertainty.^11^ The diagnostic accuracy of GPs’ clinical judgement is moderate, with a sensitivity of 58% and specificity of 89%,^12^ consistent with existing underdiagnosis of dementia in primary care.^13^ This is likely a direct consequence of the earlier mentioned barriers, leading to reluctance to communicate an impactful dementia diagnosis even though GPs have a high suspicion.

Furthermore, GPs report that the availability of ancillary investigations, such as magnetic resonance imaging (MRI) or neuropsychological testing, and pharmacological treatments is an important reason for referral.^14^ However, the National Institute for Health and Care Excellence (NICE) and Dutch GP and specialist guidelines recommend conducting ancillary investigations only when the diagnostic question remains unanswered after initial evaluation,^3,4,10^ questioning the necessity to perform these tests in most patients. This is further supported by the high practice variation among hospitals using ancillary investigations, which appears to depend more on the hospital than patient characteristics.^15,16^ Similarly, pharmacological treatments have limited effectiveness, restricting their use to secondary care.^17–19^

While previous studies have mainly focused on GPs’ perceived barriers and poor diagnostic accuracy, we hypothesise that GPs may know more about a patient’s cognitive performance than their mentioned barriers suggest and diagnostic accuracy studies are able to show, and that this implicit knowledge may be captured in GP referral letters to MCs. Thus, the aim of this study was to compare diagnostic information in GP referral letters of patients with and without eventual dementia diagnosis.

## Method

### Design and participants

This explorative, retrospective, observational study used electronic health record (EHR) data from patients visiting the Radboud University Medical Center academic geriatric MC in the Netherlands. The Strengthening the Reporting of Observational Studies in Epidemiology (STROBE) guidelines were used in the conduct and reporting of this study.^20^

We included patients aged ≥65 years with memory complaints referred to the MC by their GP between 1 January 2016 and 28 February 2020. Our age limit aligns with guideline recommendations to refer patients aged <65 years to specialists because the differential diagnosis and prognostic and therapeutic implications differ.^3^ Patients were excluded if they: 1) were referred on behalf of or by another specialist; 2) were diagnosed with dementia before referral; 3) visited the MC for a second opinion; and 4) had ancillary investigations planned before their MC visit. If patients were referred multiple times during the inclusion period, the first MC visit was used for data extraction.

### Study outcome

The primary outcome of this study was MC diagnosis, defined as the diagnosis assessed by the MC geriatrician, in most cases after a multidisciplinary meeting with geriatricians, neurologists, and neuropsychologists. MC diagnosis was categorised into dementia, mild cognitive impairment (MCI), subjective memory complaints (SMCs), other, and inconclusive. Other diagnoses were, for example, depression or delirium. We used the diagnoses established during the initial or, if applicable, subsequent MC consultation after conducting ancillary investigations. We considered the diagnosis inconclusive if no final diagnosis was stated or patients were asked to return for a reassessment after ≥3 months.

When comparing diagnostic outcome groups, we compared people with a dementia diagnosis to all patients without dementia because our main objective was identifying people with dementia who could feasibly be diagnosed in primary care. Furthermore, MCI is not considered a primary care diagnosis according to the Dutch GP and NICE guidelines.^3,21^

### Diagnostic information and patient characteristics

We collected diagnostic information from GP referral letters, including diagnostic workup elements and dementia criteria. GP diagnostic workup elements included a patient’s history, collateral history, physical examination, neurological examination, cognitive screening test, and differential diagnosis, which were scored as present or absent. Similarly, we scored the presence of dementia criteria as formulated in the Dutch GP dementia guidelines, based on the National Institute on Aging and Alzheimer's Association (NIA–AA) criteria (see Table 1).^3^

**Table 1.** Diagnostic dementia criteria formulated in the Dutch GP dementia guidelines, translated from Dutch to English

Cognitive or behavioural symptoms that:
1. Interfere with daily functioning
2. Represent a decline from previous levels of functioning and performing
3. Are not explained by delirium or depression
4. Are diagnosed based on (collateral) history-taking and objectified by a cognitive test (MMSE and clock-drawing test or RUDAS)
5. Involve ≥2 of the following domains: Impaired ability to acquire and remember new information Impaired reasoning and handling of complex tasks; poor judgement Impaired visuospatial abilities Impaired language functions Changes in personality or behaviour

MMSE = Mini-Mental State Examination. RUDAS = Rowland Universal Dementia Assessment Scale.

We collected EHR data to study how often ancillary investigations were performed at the MC. We included neuroimaging (MRI or computed tomography [CT] scan), neuropsychological assessment, consultation with an occupational therapist to assess interference in daily functioning, and lumbar puncture as ancillary investigations. We did not evaluate electroencephalogram and nuclear imaging since these have a minimal role (<1% of cases) in the diagnostic workup in this geriatric MC.

Using referral letters and EHR data, we collected patient characteristics, including demographics, morbidity, and medication use. Education level was categorised into low (1–3), middle (4 or 5), or high (6 or 7), according to the Verhage levels.^22^

### Data collection

Data extraction was performed by DR and three research interns (LN, DR, and SB). DR and two interns independently extracted data from the first 10 patient records and discussed differences to increase inter-rater agreement. We created a codebook with variable definitions for all study outcomes through extensive discussion, which we further refined during data collection. If a variable definition was changed or a category was added, we returned to earlier records to adjust them accordingly. In case of remaining uncertainty or disagreement between researchers, the GP researcher’s (MP) opinion was decisive.

### Data analysis

Descriptive statistics were used to analyse frequencies and means of patient characteristics for all diagnostic outcomes. To compare diagnostic information of patients with and without dementia, we performed χ^2^ tests, Mann–Whitney U tests, and non-paired t-tests when appropriate. All analyses were performed using IBM SPSS Statistics (version 28), and *P*-values ≤0.05 were considered statistically significant.

## Results

**Figure 1.**
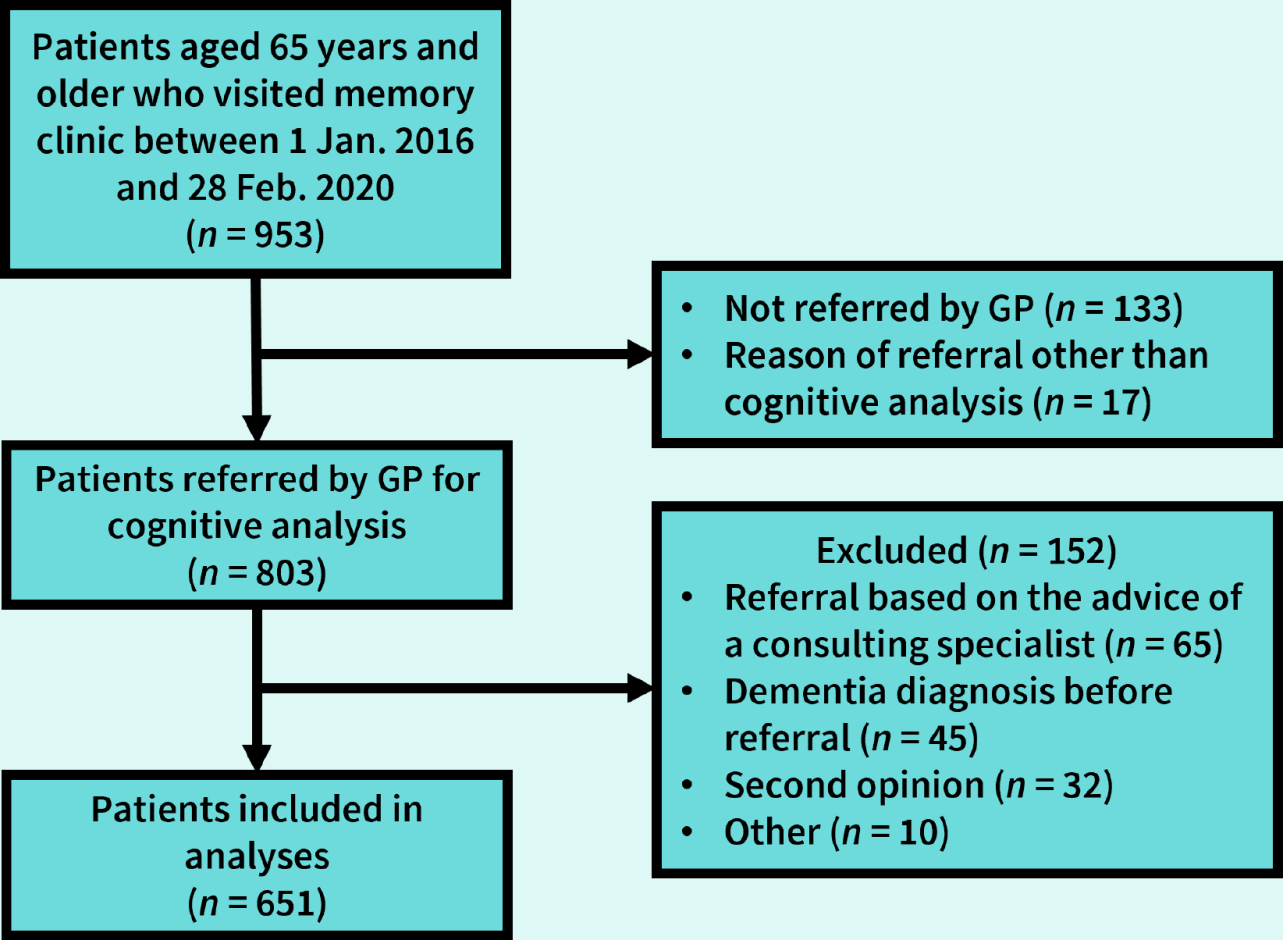
Inclusion flowchart. Reasons of referral other than cognitive analysis included analysis of functional decline (*n* = 4), functional decline and falls (*n* = 2), treatment advice for dementia, mild cognitive impairment, or persistent acoustic hallucinations (*n* = 3), and other (*n* = 8).

Within the study period, 953 patients visited the MC, of whom 803 were referred by their GP for cognitive analysis, and 152 patients were excluded for various reasons, resulting in 651 patients included in the analyses (Figure 1).

In total, 348 patients (53.5%) were diagnosed with dementia, 156 (24.0%) with MCI, 71 (10.9%) with SMCs, 26 (4.0%) with another diagnosis, and in 50 patients (7.7%) the diagnosis was inconclusive (Table 2).

**Table 2.** Characteristics of patients by different diagnostic outcome groups^a^

Characteristic	Total, *N* = 651	Dementia, *n* = 348	MCI, *n* = 156	SMCs, *n* = 71	Inconclusive, *n* = 50	Other, *n* = 26
Age, years, mean (SD)	78.0 (6.8)	79.8 (6.6)	76.8 (5.9)	73.5 (6.8)	77.6 (6.1)	73.8 (7.4)
Sex, female	348 (53.5)	199 (57.2)	77 (49.4)	28 (39.4)	31 (62.0)	13 (50.0)
Education level						
Low Moderate High Unknown	126 (19.4) 283 (43.5) 186 (28.6) 56 (8.6)	82 (23.6) 162 (46.6) 77 (22.1) 27 (7.8)	19 (12.2) 64 (41.0) 58 (37.2) 15 (9.6)	8 (11.3) 23 (32.4) 32 (45.1) 8 (11.3)	14 (28.0) 23 (46.0) 10 (20.0) 3 (6.0)	3 (11.5) 11 (42.3) 9 (34.6) 3 (11.5)
Marital status						
Married Divorced Widow(er) Other Unknown	341 (52.4) 45 (6.9) 201 (30.9) 60 (9.2) 4 (0.6)	176 (50.6) 15 (4.3) 132 (37.9) 25 (7.2) 0 (0.0)	85 (54.5) 10 (6.4) 39 (25.0) 20 (12.8) 2 (1.3)	48 (67.6) 6 (8.5) 6 (8.5) 10 (14.1) 1 (1.4)	18 (36.0) 12 (24.0) 17 (34.0) 3 (6.0) 0 (0.0)	14 (53.8) 2 (7.7) 7 (26.9) 2 (7.7) 1 (3.8)
Living situation						
Alone With others Other Unknown	280 (43.0) 352 (54.1) 14 (2.2) 5 (0.8)	164 (47.1) 176 (50.6) 7 (2.0) 1 (0.3)	62 (39.7) 88 (56.4) 5 (3.2) 1 (0.6)	18 (25.4) 51 (71.8) 0 (0.0) 2 (2.8)	23 (46.0) 24 (48.0) 2 (4.0) 1 (2.0)	13 (50.0) 13 (50.0) 0 (0.0) 0 (0.0)
Receives home care						
Yes No Unknown	142 (21.8) 435 (66.8) 74 (11.4)	99 (28.4) 224 (64.4) 25 (7.2)	20 (12.8) 111 (71.2) 25 (16.0)	4 (5.6) 56 (78.9) 11 (15.5)	14 (28.0) 30 (60.0) 6 (12.0)	5 (19.2) 14 (53.8) 7 (26.9)
Receives informal care						
Yes No Unknown	389 (59.8) 80 (12.3) 182 (28.0)	271 (77.9) 24 (6.9) 53 (15.2)	61 (39.1) 29 (18.6) 66 (42.3)	11 (15.5) 17 (23.9) 43 (60.6)	35 (70.0) 5 (10.0) 10 (20.0)	11 (42.3) 5 (19.2) 10 (38.5)
Comorbidities						
Total, mean (SD) History of depression	3.4 (2.1) 85 (13.1)	3.4 (2.1) 34 (9.8)	3.3 (2.6) 19 (12.2)	2.9 (2.0) 15 (21.1)	3.5 (2.0) 8 (16.0)	3.5 (1.9) 9 (34.6)
Total number of medications, mean (SD)	5.0 (3.7)	5.2 (3.6)	4.9 (3.9)	3.9 (3.1)	5.9 (3.8)	5.6 (4.4)

Data are presented as *n* (%) unless otherwise stated. MCI = mild cognitive impairment. SD = standard deviation. SMCs = subjective memory complaints.

Of all patients, 416 (63.9%) were diagnosed without ancillary investigations (data not shown). Ancillary investigations were performed less frequently in patients with dementia than in those without dementia (59.7% versus 67.5%, *P* = 0.039, Table 3). Ancillary investigations conducted in people with dementia were MRI (*n* = 78/348, 22.4%), neuropsychological assessment (*n* = 21, 6.0%), consultation of an occupational therapist (*n* = 21, 6.0%), CT (*n* = 7, 2.0%), and lumbar puncture (*n* = 2, 0.6%).

**Table 3.** Presence of diagnostic workup elements in GP referral letters by diagnostic outcome

Workup element	Dementia, *n* = 348	No dementia, *n* = 303	*P* value	Dementia without AI, *n* = 235	Dementia with AI, *n* = 113	*P* value
Patient’s history	305 (87.6)	277 (91.4)	0.119	205 (87.2)	100 (88.5)	0.738
Collateral history	278 (79.9)	175 (57.8)	<0.001	188 (80.0)	90 (79.6)	0.939
Physical exam	98 (28.2)	60 (19.8)	0.013	69 (29.4)	29 (25.7)	0.473
Neurological exam	26 (7.5)	29 (9.6)	0.337	11 (4.7)	15 (13.3)	0.004
Blood test	94 (27.0)	75 (24.8)	0.512	66 (28.1)	28 (24.8)	0.515
DD dementia mentioned	197 (56.6)	117 (38.6)	<0.001	137 (58.3)	60 (53.1)	0.359
MMSE performed	149 (42.8)	103 (34.0)	0.021	98 (41.7)	51 (45.1)	0.545
MMSE, performed score, mean (SD)	23.7 (3.9)	25.7 (3.3)	<0.001	23.4 (4.1)	24.3 (3.3)	0.210
Time to referral, months, mean (SD)^a^	6.0 (13.3)	3.8 (8.9)	0.003	6.1 (13.6)	5.8 (12.7)	0.893

Data are presented as *n* (%) unless otherwise stated. ^a^Time from first consultation to referral based on first contact mentioned in referral letter and referral letter date. AI = ancillary investigations. DD = differential diagnosis. MMSE = Mini-Mental State Examination. SD = standard deviation.

### Patient characteristics by diagnostic outcome

The mean age was 78.0 years (standard deviation [SD] 6.8), with a higher mean age for patients with dementia (79.8 years, SD 6.6) than patients with MCI (76.8 years, SD 5.9) and SMCs (73.5 years, SD 6.8). People with dementia received less education, were more often widowed (37.9% versus 22.8%), more often living alone (47.1% versus 38.3%), receiving informal care more often (77.9% versus 38.9%), and received home care more often (28.4% versus 14.2%) compared with people without dementia. Table 2 shows patient characteristics by diagnostic outcome.

### Diagnostic workup in GP referral letters

In GP referral letters of people with dementia, a collateral history, physical examination, differential diagnosis (DD) including dementia, and an MMSE score were more often mentioned compared with those not diagnosed with dementia (Table 3). A neurological examination was more often mentioned in referral letters of people diagnosed with dementia who had undergone ancillary investigations compared with people with dementia who had not undergone ancillary investigations. We found no other significant differences between people with dementia with and without ancillary investigations.

### Diagnostic dementia criteria in GP referral letters

In letters of people with dementia, the diagnostic criteria interference with daily functioning, a decline in functioning, and cognitive impairment in ≥2 cognitive domains were described more often than in letters of patients without dementia. In people with dementia who did not undergo ancillary investigations, interference with daily functioning was mentioned more frequently than in people with dementia who underwent ancillary investigations (Table 4).

**Table 4.** Presence of diagnostic dementia criteria in GP referral letters by diagnostic outcome

Diagnostic dementia criterium	Dementia, *n* = 348	No dementia, *n* = 303	*P* value	Dementia without AI, *n* = 235	Dementia with AI, *n* = 113	*P* value
Symptoms interfere with daily functioning	152 (43.7)	78 (25.7)	<0.001	112 (47.7)	40 (35.4)	0.031
Symptoms represent decline from previous levels of functioning	232 (66.7)	154 (50.8)	<0.001	159 (67.7)	73 (64.6)	0.571
Symptoms not explained by delirium or depression	7 (2.0)	7 (2.3)	0.618	1 (0.4)	6 (5.3)	N/A^a^
Symptoms diagnosed based on history-taking and cognitive test	71 (20.4)	30 (9.9)	0.002	47 (20.0)	24 (21.2)	0.788
Cognitive impairment in ≥2 domains	219 (62.9)	133 (43.9)	<0.001	218 (92.8)	106 (93.8)	0.720
≥2 diagnostic criteria present	217 (62.4)	125 (41.3)	<0.001	148 (63.0)	69 (61.1)	0.730
≥3 diagnostic criteria present	130 (37.4)	49 (16.2)	<0.001	93 (39.6)	37 (32.7)	0.217

Data are presented as *n* (%) unless otherwise stated. ^a^N/A = not applicable; groups too small for statistical testing, *n* = 7. AI = ancillary investigations.

**Figure 2.**
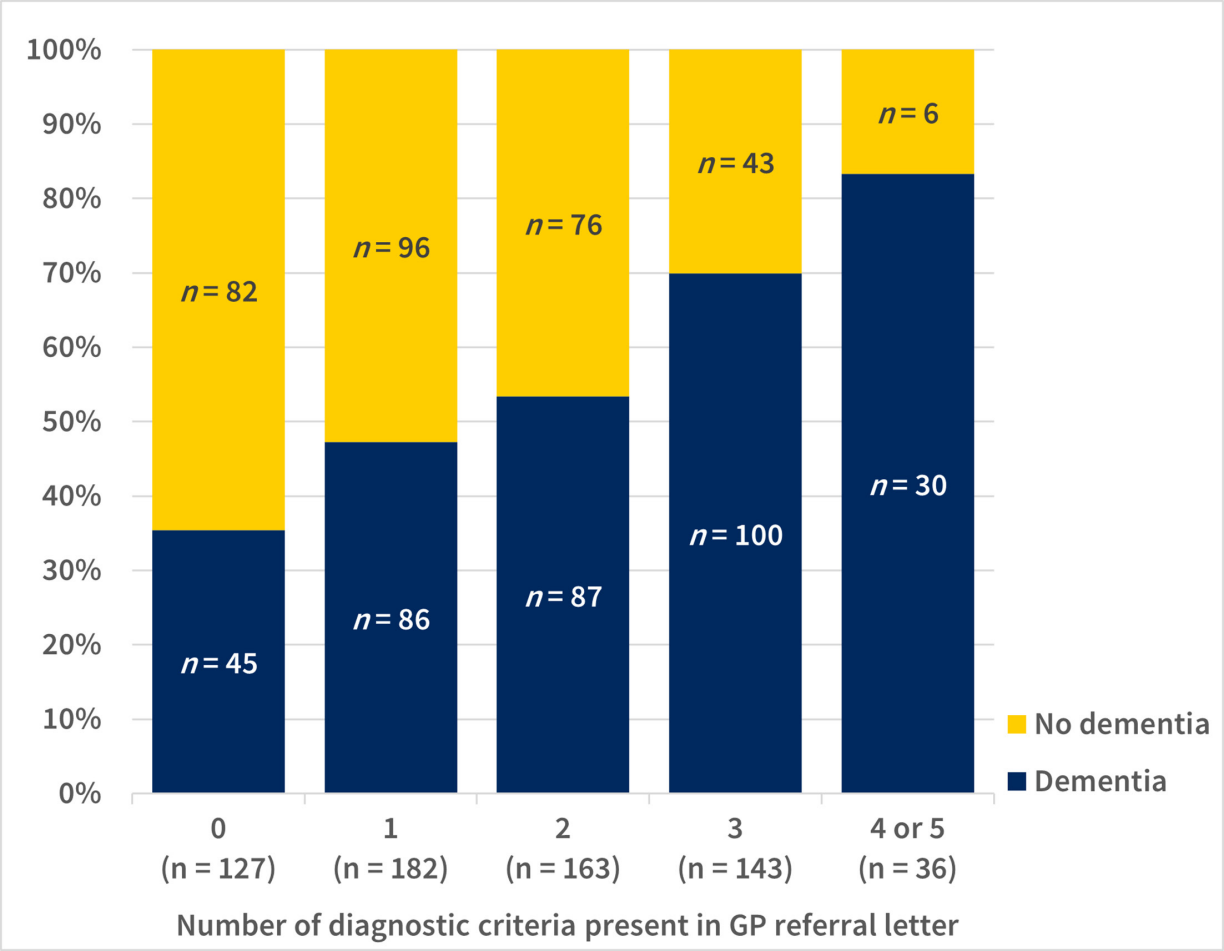
Proportion of people diagnosed with dementia by number of diagnostic criteria present in GP referral letters

With each additional diagnostic criterion mentioned in the GP referral letter, the chance of being diagnosed with dementia in the MC increased, starting at 35.4% for patients with no criteria, 47.3% for those with one criterion, 53.4% for two criteria, 69.9% for three criteria, and reaching 83.3% for patients with four or five criteria according to the referral letter (*P*<0.001, Figure 2). The number of diagnostic criteria was not associated with whether ancillary investigations were performed in people with dementia (*P* = 0.515).

## Discussion

### Summary

In people with memory complaints referred by their GP to a Dutch academic geriatric MC, dementia was often diagnosed without the use of ancillary investigations. GPs more often mentioned different diagnostic workup elements and dementia criteria in the referral letters of people who were diagnosed with dementia at the MC than in people without dementia. The more dementia criteria GPs mentioned in the referral letter, the more likely a person was diagnosed with dementia.

These findings suggest that GPs already have a strong suspicion of dementia in these patients eventually diagnosed with dementia at the MC and that these patients could have been diagnosed with dementia in primary care, as there was no need for diagnostic tools that are unavailable in primary care. These insights shed new light on dementia diagnosis in primary care, as previous research tended to focus on GP barriers to dementia diagnoses and the moderate diagnostic accuracy of GP diagnoses.

### Strengths and limitations

This study provides novel insights into current practices and clinical reasoning of GPs by collecting data from referral letters. One of the strengths of this study is that it reflects clinical practice by using routinely collected data in a representative older population, thereby warranting the generalisability of our results for the primary care population. Furthermore, this study included a large group of patients.

A limitation of this study is that the content of the referral letters varied greatly, ranging from nearly empty to very rich in information. Empty referral letters lacked diagnostic workup and criteria data, limiting insight into the GP’s clinical reasoning. Time constraints and the lack of relevant information to be mentioned may be explanations for this besides a lack of knowledge. This study was conducted in a single academic MC, which may limit the generalisability of our results because the referred patient population may be less representative of general MCs in community hospitals. However, the mean age and sex distribution of the patients in our study were consistent with those observed in a primary care cohort of people with memory complaints^23^ and other regular MC cohorts.^5,24–26^ The relative distribution of diagnoses (dementia, MCI, SMC, and other) was similar to the average of 78 MCs in the Netherlands,^9^ suggesting that our study population is likely to represent the average primary care population of referred people with memory complaints.

The judgement of the presence of workup and diagnostic criteria in referral letters was based on free text and, therefore, an interpretation of the researchers. We tried to overcome this limitation by frequently consulting with each other during data extraction, adhering to guideline terms as much as possible, and noting coding agreements.

### Comparison with existing literature

Our study results feed the hypothesis that GPs often already strongly suspect on referral whether their patients have dementia or not. A recent systematic review, including diagnostic clinical judgement studies in primary care, reported a moderate diagnostic accuracy and the tendency to underdiagnose dementia.^12^ However, the overall sensitivity for cognitive impairment was higher, with a somewhat lower specificity. An explanation for this may be the hypothesis supported by our study results that GPs often know that there is cognitive impairment but are hesitant to 'label' a patient with dementia.

Just under 40% of referral letters included a cognitive test, despite GP guidelines recommending performing a cognitive screening test before referral. Previous studies mainly reported lower rates ranging from 13.2%–41.3%,^27–29^ with an increasing trend over time. GPs indicate a need for a good cognitive test but appear to perform a guideline-based cognitive test in less than half of their patients. This could have several explanations such as time constraints, difficulty with test score interpretation, or already planning to refer the patient regardless of test outcome.

### Implications for research and practice

Our results suggest that most patients who are currently referred to MCs could be diagnosed in primary care. This is in line with recommendations in the Dutch and UK dementia guidelines. Our results could enhance GPs’ awareness and confidence in diagnosing patients in primary care when no clear indication for referral is present, such as rapidly progressive dementia, early onset dementia, or focal deficits on neurological examination. Following the guidelines more closely, GPs could check how many criteria for dementia are fulfilled and decide not to refer if this is, for instance, ≥4, because this will likely lead to a dementia diagnosis in an MC. This approach ensures accessibility of specialist services, particularly given the increasing number of people with dementia.

In addition to diagnostic uncertainty, GPs may refer patients to other professionals for diagnosis to avoid damaging their longstanding positive doctor–patient relationship or owing to time constraints.^30–33^ Since ancillary investigations at an MC and thereby visiting an MC are often unnecessary, innovative collaboration models between primary care and MCs could offer a solution. For example, an MC specialist could assist the GP remotely, thus eliminating the need for an MC visit. Similarly, an elderly care physician, a Dutch physician who follows a 3-year specialist training course to care for older people,^34^ could provide direct consultation in primary care. These approaches could not only help in addressing diagnostic uncertainty but also in involving an external party for diagnosis, thereby preserving the doctor–patient relationship. Post-diagnostic care in the Netherlands is already primarily organised by primary care professionals, and is both cost-effective and of comparable quality with care organised by MCs.^35,36^

If our findings were to result in an increase in primary care diagnoses and a decrease in referrals, this may sometimes lead to delayed diagnoses. The question is whether that is wrong or problematic, because, currently, there are no effective treatments that can delay or stop further meaningful cognitive decline.^37^ The decision to start a diagnostic trajectory is considered preference based,^38,39^ and a timely diagnosis is not the same as an early diagnosis,^40^ implying that factors beyond the previously studied diagnostic accuracy of GPs are important. Diagnostic processes within primary care offer advantages, such as the patient’s familiarity with a healthcare provider who understands their context well. Conversely, referrals can have downsides, including the burden of visiting a hospital and the potential for incidental findings. To compare primary and secondary care diagnostic trajectories, we have initiated a trial using daily functioning as the primary outcome measure.^41^

## Data

The dataset relied on in this article is available from the corresponding author on reasonable request.
